# Endothelial Cell Changes After Pars Plana Vitrectomy: A Cross-Sectional Study in a Tertiary Care Center

**DOI:** 10.7759/cureus.73250

**Published:** 2024-11-07

**Authors:** Irfan Akber Malik, Kashif Iqbal, Jawad Bin Yamin Butt, Muhammad Farhan Lodhi, Saad Muhammad Iqbal, Faisal Iqbal, Mohammad Haroon Khalid, Hafiz Habib Ur Rehaman Khalil

**Affiliations:** 1 Ophthalmology, Layton Rahmatullah Benevolent Trust (LRBT) Hospital Township, Lahore, PAK; 2 Ophthalmology, Akhtar Saeed Medical and Dental College, Lahore, PAK; 3 Ophthalmology, Sargodha Medical College, Sargodha, PAK; 4 Gastroenterology, Lahore General Hospital, Lahore, PAK

**Keywords:** corneal endothelium, endothelial cell density, ophthalmic surgery, pars plana vitrectomy, specular microscopy

## Abstract

Background: A wide variety of vitreoretinal diseases have been majorly treated by the use of pars plana vitrectomy (PPV) as the most commonly practiced treatment option. Despite the fact that it is the most feasible treatment modality, the probability of corneal endothelial cell damage following the treatment remains a matter of great concern. The study aims to inquire about the aftermath of PPV on endothelial cell density (ECD) and spans a time period of six months after the surgery has been performed.

Methodology: This cross-sectional study involved 20 patients who underwent PPV at Layton Rahmatullah Benevolent Trust (LRBT) Hospital Township, Lahore, Pakistan. ECD was measured using non-contact specular microscopy preoperatively and at 15 days, two months, and six months postoperatively. The main outcome measured was the change in ECD over time, and the results were analyzed using descriptive statistics via SPSS software (version 25.0, IBM Corp., Armonk, NY).

Results: The mean baseline ECD was 2,421 cells/mm², which decreased to 2,185 cells/mm² by the six-month follow-up, representing an overall mean ECD loss of 13.72%. Significant reductions in ECD were observed at each postoperative time point, with the most substantial decrease occurring within the first two months post-surgery.

Conclusion: Corneal endothelial cells are substantially influenced by PPV, which is significantly evident by the continuous reduction in ECD following surgery. The analysis calls attention to a careful and proper post-operative observation of corneal well-being is therefore required to alleviate likely endothelial damage along with the necessity of deliberate thinking while opting for the surgical techniques. The main focus of future studies should be on how to improve the current practices involving surgical procedures to ensure minimizing the loss of endothelial cells.

## Introduction

Many vitreoretinal diseases have been treated with pars plana vitrectomy (PPV), a surgical procedure most commonly practiced [1]. The most favorable reason for which this surgical procedure is employed, is the potential of the treatment to reinstate the patient’s vision while addressing the basic vitreoretinal issues, providing an immediate and efficacious treatment option for all the conditions that will otherwise lead to severe consequences such as vision impairment or blindness [2]. In the procedure of PPV, minute incisions are made in the eye’s ciliary body known as pars plana, from where the operating surgeon gains access to vitreous humor and retina in order to make required corrections or to remove any opacities in the vitreous [3].

PPV holds several potential hazardous effects for the corneal endothelium regardless of its extensive use and humongous success in clinical aspects. Corneal hydration and transparency are critically maintained by the corneal endothelium. Corneal edema and loss of sharp sight can occur as a result of damage to endothelial cells during PPV, and this could be a serious postoperative challenge [4,5].

Mechanical trauma caused by surgical instruments, fluctuation in the intraocular pressure, and exposure to air or gas during the procedure, are some of the important factors that could be the cause of the vulnerability of endothelium during PPV. Notably, the corneal function might be compromised in the long term postoperatively due to decreased endothelial cell density (ECD) [5,6]. In this regard, different research has specified various extents of endothelial cell detriment across different patient populations and among different surgical techniques that were employed. All of these focus the attention on better and in-depth comprehension of these dynamics [7,8].

The hypothesis of this current investigation is to address the significant information gap in the context of the magnitude and consequences of endothelial cell damage followed by PPV. Former research studies in this regard have not been able to find well-established connections between various surgical procedures and the after-effects on the endothelium nor were they able to list down the specific risk factors associated with the procedures. In addition to this, some of the researchers have pin-pointed potentially safe and protective surgical strategies including some modifications in surgical techniques or using specific surgical accessories, all of these adaptations are short of authentic and invariable supporting data [8,9].

The main purpose of this study is to meticulously assess the changes in the density and morphology of endothelial cells following PPV in a tertiary care setup. The objectives of the present research include quantification of the extent of endothelial cell loss, identification of the probable risk factors that are likely to be associated with the loss, and inspection of the association of a patient’s quality of life with visual acuity after the surgical procedure is performed. A better understanding of the above is of great significance in the proceeding surgical techniques, improvement in patient outcomes along with reducing the chances of postoperative complexities which are therefore associated with PPV [1,8].

The objectives of the study were achieved by employing a cross-sectional design, the patients enrolled in the study underwent PPV at a tertiary care center. Pre and postoperative ECD will be calculated with the help of specular microscopy, which is considered to be an authentic technique in order to evaluate the morphology and density of endothelial cells. A detailed analysis regarding the agility of the endothelial cells both, before and after the surgical procedure will be at hand. This will also allow for precise evaluation of the cell loss that is accountable to the surgical procedure [5,7].

Moreover, this study also includes an extensive review of some records from the surgical procedures in order to correspond to particular intraoperative circumstances and techniques with the postoperative consequences of endothelial cells. Risk factors that can be modified alongside possibly safe surgical practices will also be identified with the help of this review. The explorations would definitely provide applicable knowledge that can be easily merged into the ongoing clinical practices which will possibly result in improving the existing surgical techniques and also enhancement of counseling and management policies for the patients [6,9].

The present study bridges an essential discrepancy in the existing literature on ophthalmology by rendering an in-depth examination of how PPV influences corneal endothelial cells. The significance of the study is quite extensive, presenting the prospective to strengthen the surgical protocols as well as the outcomes related to patients remarkably with regards to vitreoretinal surgery. With the awareness of endothelial damage and the involved mechanisms and outcomes that are linked to this, ophthalmologists will then be able to work on increasing the overall safety and efficiency of PPV and also reduce the associated risks.

The primary objective of this study is to explore endothelial cell damage following PPV and to analyze the factors that might influence this outcome. By establishing a clearer link between PPV and endothelial cell dynamics, this study aims to contribute to safer surgical practices and improved patient outcomes in terms of visual acuity and corneal health.

## Materials and methods

Study design and setting

This cross-sectional study was designed to explore endothelial cell damage in patients undergoing PPV. The research was conducted at Layton Rahmatullah Benevolent Trust (LRBT) Hospital Township, Lahore, Pakistan. A total of 20 patients who underwent PPV were enrolled to evaluate the changes in their corneal endothelial cells postoperatively.

Participants

Individual adults 18 years and above who underwent PPV, and who were diagnosed for either retinal detachment, macular holes, or vitreous hemorrhages at LRBT Hospital were all included in the study. The exclusion criteria set for the study participants were those patients who had pre-existing endothelial dystrophies, prior corneal surgeries, or miscellaneous ocular conditions like glaucoma or anterior segment surgeries, that are already known to influence the ECC. All of the participants included had the surgery performed on one of their eyes, in total 20 eyes that were affected were studied altogether in this study. 

Data collection

Preoperative and postoperative examinations were scheduled. Preoperative data collection included a comprehensive ophthalmic examination: visual acuity, intraocular pressure measurement, slit-lamp examination, and dilated fundus examination. ECD was measured using non-contact specular microscopy (Topcon SP-2000P; Topcon America Corp, Paramus, NJ) one day before the surgery and then at 15 days, two months, and six months postoperatively to assess the endothelial cell loss over time.

Surgical procedure

All PPV surgeries were performed by the same experienced retinal surgeon to minimize variability in surgical technique. The standard 23-gauge three-port PPV technique was employed. Surgical indications and any intraoperative complications were recorded meticulously. The surgical technique involved removing the vitreous humor and treating the underlying retinal pathology without the use of intraocular tamponades such as silicone oil, which could influence ECCs.

Outcome measures

The primary outcome measure was the change in ECD from the preoperative baseline to the postoperative follow-ups. Secondary outcome measures included visual acuity changes and any postoperative complications directly relating to the surgery. Endothelial cell loss was calculated as the percentage decrease in ECD from baseline at each follow-up period.

Statistical analysis

Descriptive statistics were used to summarize patient demographics and clinical characteristics. Mean best corrected visual acuity (BCVA) (LogMar), ECD (cells/mm²) at baseline, post-six months, and percentage of ECD loss in phakic eyes (%), and pseudophakic eyes (%) were presented in tabular form. All analyses were performed using SPSS software (version 25.0, IBM Corp., Armonk, NY).

Ethical considerations

The study protocol was approved by the Institutional Review Board (IRB) of LRBT Hospital Township. All participants provided written informed consent after being informed about the nature of the study, the procedures involved, potential risks, and benefits. The study adhered to the tenets of the Declaration of Helsinki and ensured the confidentiality and anonymity of the participants.

## Results

A total of 20 participants with a mean age of 57.2 ± 9.0 years were included in the study. There was equal distribution of gender. Comorbid conditions were prevalent, with six (30%) of the participants having diabetes mellitus and seven (35%) of the participants having hypertension, reflecting common health issues in the target age group. Eye status was equally divided between phakic and pseudophakic as illustrated in Table 1.

**Table 1:** Sociodemographic and clinical profile of study patients

Variable	Details
Total participants	20
Age (years) mean ± SD	57.2 ± 9.0
Gender distribution	
Male	10 (50%)
Female	10 (50%)
Comorbidities	
Diabetes mellitus	6 (30%)
Hypertension	7 (35%)
None	7 (35%)
Eye status	
Phakic	10 (50%)
Pseudophakic	10 (50%)

**Figure 1:**
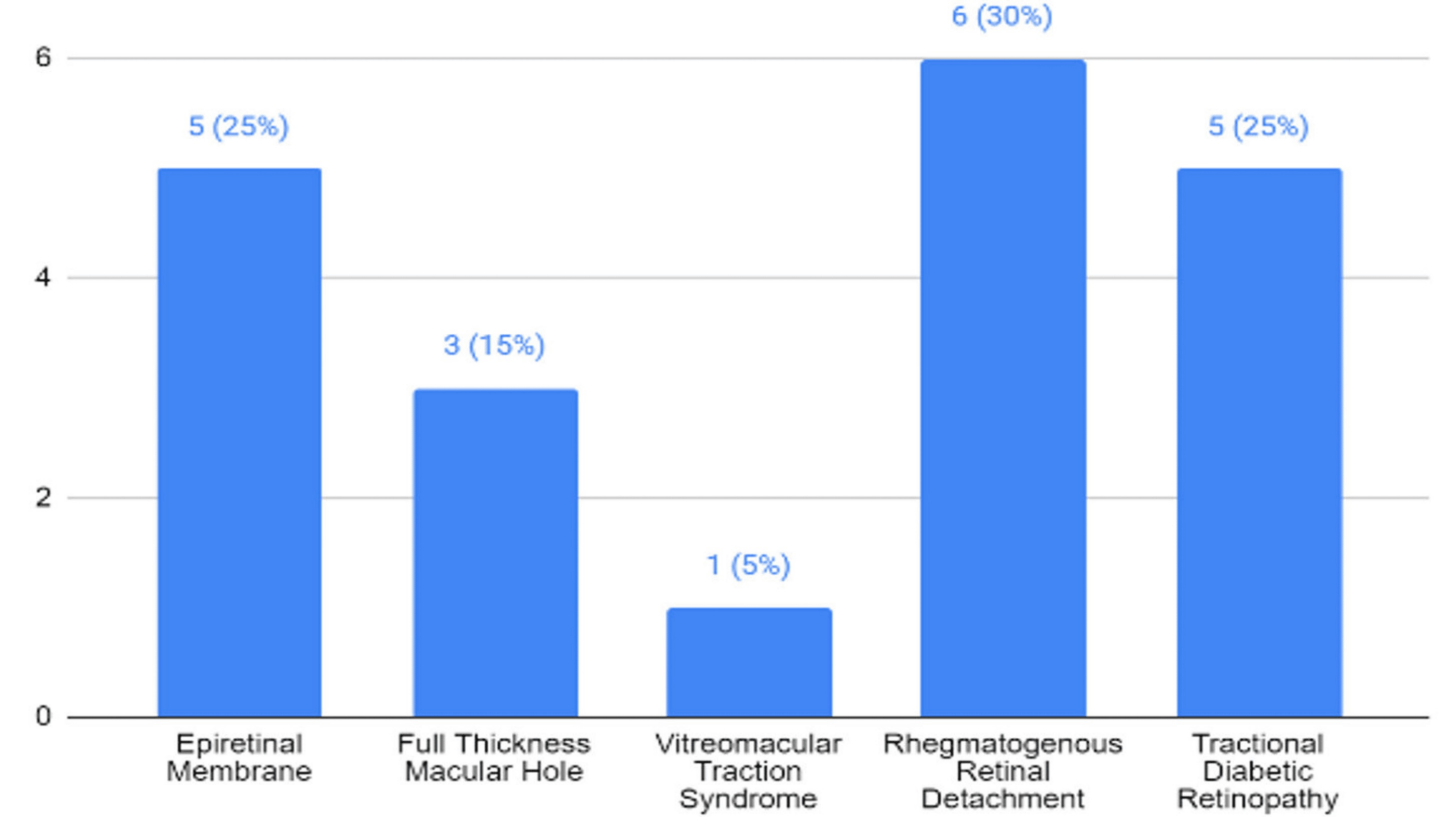
Distribution of diagnosis among patients (n=20)

The diagnosis distribution among the participants undergoing PPV reveals a balanced mix of vitreoretinal conditions, with rhegmatogenous retinal detachment (RRD) and epiretinal membrane (ERM) being the most common, each constituting six (30%) and five (25%) of the cases, respectively. Full-thickness macular hole (FTMH), vitreomacular traction (VMT) syndrome, and tractional diabetic retinopathy (TDR) collectively account for the remaining 45%, indicating a diverse set of indications for vitrectomy within this patient cohort (Figure 1).

In this cross-sectional study involving 20 participants undergoing PPV at LRBT Hospital, Lahore, the analysis revealed a mean BCVA of 0.61 LogMar at baseline, indicating moderate visual impairment. The ECD showed a progressive decline from an average baseline value of 2,421 cells/mm², decreasing to 2,268 cells/mm² at 15 days, 2,197 cells/mm² at two months, and 2,185 cells/mm² at six months postoperatively. This gradual reduction in ECD reflects the expected endothelial cell loss following vitrectomy, with an overall mean decrease of 13.72% over the six months. This decrease is consistent across both phakic and pseudophakic eyes, though phakic eyes exhibited slightly higher percentage losses (14.80%) compared to pseudophakic eyes (13.62%) (Table 2).

**Table 2:** Clinical outcomes among patients who underwent pars plana vitrectomy BCVA - Best Corrected Visual Acuity, ECD - Endothelial cell density

Clinical variables	Mean ± SD
Mean BCVA (LogMar) at baseline	0.61 ± 0.27
Mean ECD at baseline (cells/mm²)	2,421 ± 179
Mean ECD post-6 months (cells/mm²)	2,185 ± 160
Mean ECD loss at 6 months (%)	13.72 ± 2.84
Percentage ECD loss in phakic eyes (%)	14.80
Percentage ECD loss in pseudophakic eyes (%)	13.62

## Discussion

In this study, we focused on 20 patients who were treated with PPV at a tertiary care hospital, and alterations in the ECD were observed following the surgical procedure. PPV as a surgical approach revealed an alarming trend of continuous endothelial cell loss over the period although it is considered an effective treatment option for a wide range of vitreoretinal disorders. A mean reduction in ECD of about 13.72% was detected over a span of six months, which led to some doubts regarding the long-term significance of the surgery on corneal health.

The findings of our study are in accordance with the previous research where Chicharro et al. [10-13] reported similar endothelial losses and supported the fact that PPV can substantially affect the strength of endothelial cells. Moreover, research findings from Goezinne et al. [14] also confirm our reflection through the demonstration of endothelial cell loss after vitrectomy, specifically when silicon oil is being used as a tamponade. Additional endorsement of the fact is provided by Ibrahim et al. [15], who reported the reduction in ECD following vitrectomy and advocated a need for potentially remodeled surgical procedures along with comprehensive awareness.

Nonetheless, all the studies were not on the same page. Contrary to our findings, Coman et al. [16] reported no significant changes in ECD following PPV, suggesting that specific surgical techniques and patient demographics might influence endothelial cell outcomes. Similarly, Confalonieri et al. [17] observed minimal changes in ECD, attributing endothelial cell survival to factors such as the type of tamponade used and the duration of surgery. Minami [18] emphasized that even slight alterations in endothelial cells suggest a need for alternative approaches in PPV treatment, which may yield different results. These discrepancies highlight the potential impact of variables such as technique, tamponade, risk factors, and patient demographics on ECD, which we recognize as important considerations for further exploration in future studies.

Meticulous attention is therefore the foremost requirement during patient selection and consideration of the appropriate surgical technique. As stated, the continuous loss of ECD that is perceived by the research findings, there is a firm need to improvise the current surgical techniques probably by reducing intraocular manipulation along with the introduction of usage of protective agents. Moreover, meticulous management of likely ramifications such as corneal edema must be dealt with strict postoperative care.

However, this particular study also has its own limitations. The generalizability of our study is questionable as we had a limited sample size and the study was only conducted in one of the Hospitals. At the same time, the use of compatible methodology approaches and precision of the measured after-effects provided the credibility to validate our findings. These particular explorations are of greater importance for ophthalmic practitioners and may hold importance in refining clinical guidelines for the future to ameliorate patient outcomes following PPV.

## Conclusions

In conclusion, this study highlights the significant impact of PPV on corneal endothelial cells, as indicated by a progressive loss of ECD. While our findings align with some previous studies and differ from others, it is clear that PPV outcomes largely depend on the chosen surgical technique and patient-specific factors. Given the advancements and improvements since PPV was first performed over 50 years ago, the procedure remains an indispensable approach for interventions requiring access to the inner eye. Further research with larger sample size and multicenter studies will be beneficial to deepen our understanding of PPV’s impact on endothelial health in light of these ongoing innovations.
